# The tripeptide *N*-Cbz-βGly-Gly-Gly-Obz

**DOI:** 10.1107/S2056989015004272

**Published:** 2015-03-14

**Authors:** Sumesh Nicholas

**Affiliations:** a Department of Physics, Indian Institute of Science, Bangalore 560012, India

**Keywords:** crystal structure, glycine, peptide, β-peptide, β-glycine,hydrogen bonding

## Abstract

The title peptide, *N*-benzyl­oxycarbonyl-β-glycylglycylglycine benzyl ester, C_22_H_25_N_3_O_6_, contains a non-proteinogenic amino acid residue, β-glycine, which is a homologated analogue of glycine. In the mol­ecular structure, β-glycine adopts an extended conformation with a *trans* conformation about its C^β^—C^α^ bond. The second glycine residue adopts an extended conformation while the third glycine residue adopts a helical conformation. In the crystal, three N—H⋯O hydrogen bonds, two involving the same carbonyl O atom as acceptor, results in an infinite two-dimensional network parallel to the *bc* plane.

## Related literature   

For comprehensive reviews on β-amino acids and β-peptides, see: Cheng *et al.* (2001 ▸); Seebach *et al.* (2004 ▸). For conformations and structural features of β-peptides, see: Appella *et al.* (1996 ▸, 1997 ▸); Seebach & Matthews (1997 ▸); Gellman (1998 ▸); Hill *et al.* (2001 ▸); Seebach *et al.* (1996 ▸, 2005 ▸, 2006 ▸). For the conformations of hybrid peptide sequences formed of α-, β- and higher ω-amino acids, see: Banerjee & Balaram (1997 ▸); Karle *et al.* (1997 ▸); Gopi *et al.* (2002 ▸); Roy & Balaram (2004 ▸); Ananda *et al.* (2005 ▸); Roy *et al.* (2005 ▸); Schmitt *et al.* (2005 ▸, 2006 ▸); Sharma *et al.* (2009 ▸); Schramm *et al.* (2010 ▸).
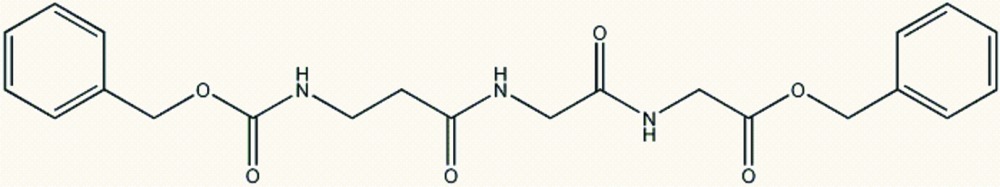


## Experimental   

### Crystal data  

C_22_H_25_N_3_O_6_
*M* *_r_* = 427.45
Monoclinic, 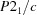

*a* = 24.713 (3) Å
*b* = 9.6794 (10) Å
*c* = 8.9445 (10) Å
β = 92.257 (5)°
*V* = 2137.9 (4) Å^3^
*Z* = 4
Mo *K*α radiation
μ = 0.10 mm^−1^
*T* = 293 K
0.4 × 0.2 × 0.04 mm

### Data collection  

Bruker Kappa APEXII CCD diffractometer
Absorption correction: multi-scan (*SADABS*; Bruker, 2001 ▸) *T*_min_ = 0.623, *T*_max_ = 0.746
17172 measured reflections
5253 independent reflections
2790 reflections with *I* > 2σ(*I*)
*R*_int_ = 0.031

### Refinement  

*R*[*F* ^2^ > 2σ(*F* ^2^)] = 0.096
*wR*(*F* ^2^) = 0.326
*S* = 1.06
5253 reflections
300 parameters
H atoms treated by a mixture of independent and constrained refinement
Δρ_max_ = 0.36 e Å^−3^
Δρ_min_ = −0.48 e Å^−3^

### 

Data collection: *APEX2* (Bruker, 2007 ▸); cell refinement: *SAINT-Plus* (Bruker, 2007 ▸); data reduction: *SAINT-Plus*; program(s) used to solve structure: *SHELXS97* (Sheldrick, 2008 ▸); program(s) used to refine structure: *SHELXL97* (Sheldrick, 2008 ▸); molecular graphics: *ORTEP-3 for Windows* (Farrugia, 2012 ▸) and *Mercury* (Macrae *et al.*, 2006 ▸); software used to prepare material for publication: *SHELXL97*.

## Supplementary Material

Crystal structure: contains datablock(s) global, I. DOI: 10.1107/S2056989015004272/lr2132sup1.cif

Structure factors: contains datablock(s) I. DOI: 10.1107/S2056989015004272/lr2132Isup2.hkl

Supporting information file. DOI: 10.1107/S2056989015004272/lr2132Isup3.docx

Supporting information file. DOI: 10.1107/S2056989015004272/lr2132Isup4.docx

**Fig. 1.**
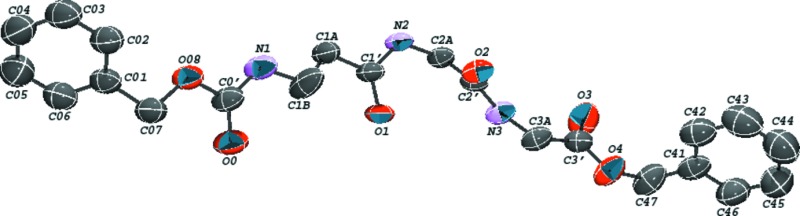
Thermal Ellipsoid plot of NCbz-β Gly-Gly-Gly-Obz drawn at 50% probability level. Hydrogen atoms have been omitted for clarity.

. DOI: 10.1107/S2056989015004272/lr2132fig1.tif
Thermal Ellipsoid plot of NCbz-β Gly-Gly-Gly-Obz drawn at 50% probability level. Hydrogen atoms have been omitted for clarity.

**Fig. 2.**
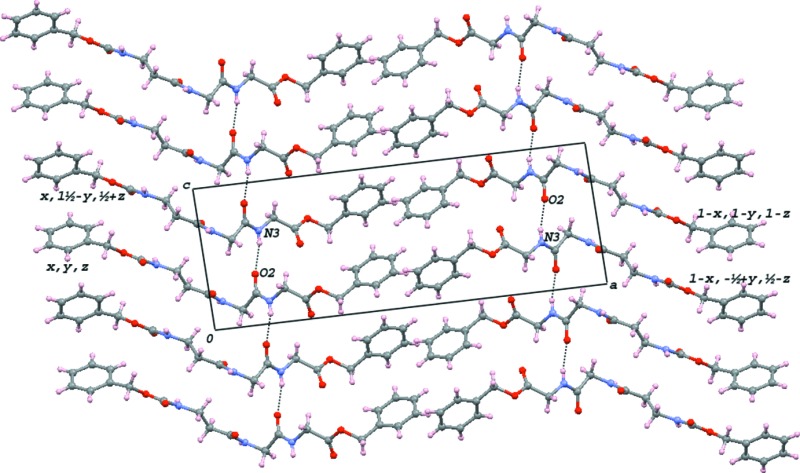
A view of the packing of NCbz-βGly-Gly-Gly-Obz as viewed down the b-axis. Intermolecular hydrogen bonds are represented as dotted lines.

. DOI: 10.1107/S2056989015004272/lr2132fig2.tif
A view of the packing of NCbz-βGly-Gly-Gly-Obz as viewed down the b-axis. Inter­molecular hydrogen bonds are represented as dotted lines.

**Fig. 3.**
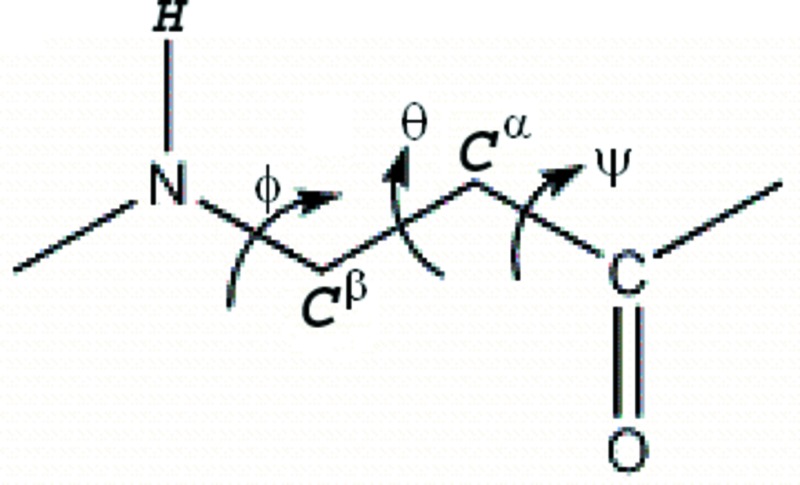
Atomic labeling and definition of backbone torsion angles in case of β-residues.

. DOI: 10.1107/S2056989015004272/lr2132fig3.tif
Atomic labeling and definition of backbone torsion angles in case of β-residues.

CCDC reference: 1051721

Additional supporting information:  crystallographic information; 3D view; checkCIF report

## Figures and Tables

**Table 1:** Hydrogen-bond geometry (, )

*D*H*A*	*D*H	H*A*	*D* *A*	*D*H*A*
N2H2O1^i^	0.83(4)	2.13(4)	2.951(3)	169(4)
N3H3O2^ii^	0.79(5)	2.11(5)	2.868(3)	161(5)
N1H1O2^iii^	0.76(5)	2.20(5)	2.959(4)	176(2)

Symmetry codes: (i) 
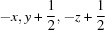
; (ii) 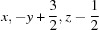; (iii) 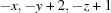.

## supplementary crystallographic information

### S1. Chemical context

Insertion of methyl­ene units to the backbone of α-amino acids generates a family of amino acids referred to as ω-amino acids (Cheng *et al.*, 2001; Seebach *et al.*, 2004). β-Amino acids are obtained when a single methyl­ene unit is added to the backbone of an α-amino acid. Compounds containing β-amino acids are ubiquitously found in biological systems (Seebach *et al.*, 2004). The simplest β-amino acid, β-glycine, is a component of co-enzyme A, pantothenic acid and carnosine. β-amino acids have an additional degree of torsional freedom about the C^β^—C^α^ bond (θ) and this increases the conformational possibilities of peptides formed of β-amino acids.

Inter­est in β-amino acids has resulted in a considerable body of work on the conformation of polypeptides formed of β-amino acids (Seebach and Matthews, 1997; Gellman, 1998; Hill *et al.*, 2001; Cheng *et al.*, 2001; Seebach *et al.*, 2004, 2005, 2006). Hybrid sequences containing α-, β- and higher ω-amino acids have also been investigated (Banerjee and Balaram, 1997; Karle *et al.*, 1997; Gopi *et al.*, 2002; Roy and Balaram, 2004; Ananda *et al.*, 2005; Roy *et al.*, 2005; Schmitt *et al.*, 2005, 2006; Sharma *et al.*, 2009; Schramm *et al.*, 2010). Helical structures formed by β-peptides have been observed by several research groups (Seebach *et al.*, 1996, 2005; Appella *et al.*, 1996, 1997).

In case of β-amino acids, information regarding the conformational preferences can only be obtained by crystallographic characterization of synthetic peptides unlike in case of α-amino acids where such information can be gathered from the crystal structures of proteins. This paper presents the crystallographic characterization of a synthetic peptide containing a β-glycine residue.

### S2. Molecular Conformation

The first two glycine residues of the peptide molecule adopt extended conformations while the third glycine residue adopts a helical conformation. βGly(1) adopts torsion angle values φ_1_ = 146.5° and ψ_1_ = -155.9°. *Trans* conformation is observed about the C^β^—C^α^ bond of the βGly(1) residue. Gly(2) adopts torsion angles φ_2_ = -61.0° and ψ_2_ = 151.4° while Gly(3) adopts torsion angle values φ_3_ = -137.2° and ψ_3_ = -170.4°. Since the crystal structure is that of an achiral peptide crystallized in a centrosymmetric space group, the choice of sign for torsion angles is arbitrary. There are no intra­molecular hydrogen bonds in the crystal structure.

### S3. Supra­molecular features

An analysis of the packing of molecules in the crystal revealed the presence of three inter­molecular hydrogen bonds. Molecules related by the symmetry (-*x*, 1/2 + *y*, 1/2 - *z*) associate through hydrogen bonds resulting in columns of hydrogen bonded molecules extending along the crystallographic *b*-direction. Aggregation also occurs *via* inter­molecular bifurcated hydrogen bonding involving a carbonyl oxygen and two donor NH groups.

### S4. Synthesis and crystallization

The title compound was purchased commercially. Plate-like crystals of the title compound were obtained by slow evaporation from methanol/water solution.

### S5. Refinement

The N-bound H atoms and H-atoms bound to C2A could be located from difference Fourier maps. The remaining C-bound H atoms were fixed geometrically in calculated positions and refined as riding atoms. During refinement, H-atoms attached to aromatic rings were positioned with C—H = 0.93 Å and *U*_iso_(H) = 1.2*U*_eq_(C) while methyl­ene H-atoms were positioned with C—H = 0.97 Å and *U*_iso_(H) = 1.2*U*_eq_(C).

### Crystal data

**Table:**

C_22_H_25_N_3_O_6_	*Z* = 4
*M**_r_* = 427.45	*F*(000) = 904
Monoclinic, *P*2_1_/*c*	*D*_x_ = 1.328 Mg m^−^^3^
Hall symbol: -P 2ybc	Mo *K*α radiation, λ = 0.71073 Å
*a* = 24.713 (3) Å	µ = 0.10 mm^−^^1^
*b* = 9.6794 (10) Å	*T* = 293 K
*c* = 8.9445 (10) Å	Platy, colourless
β = 92.257 (5)°	0.4 × 0.2 × 0.04 mm
*V* = 2137.9 (4) Å^3^	

### Data collection

**Table:**

Bruker Kappa APEXII CCD diffractometer	5253 independent reflections
Radiation source: fine-focus sealed tube	2790 reflections with *I* > 2σ(*I*)
Graphite monochromator	*R*_int_ = 0.031
φ and ω scan	θ_max_ = 29.0°, θ_min_ = 1.7°
Absorption correction: multi-scan (*SADABS*; Bruker, 2001)	*h* = −32→32
*T*_min_ = 0.623, *T*_max_ = 0.746	*k* = −12→12
17172 measured reflections	*l* = −11→11

### Refinement

**Table:**

Refinement on *F*^2^	Primary atom site location: structure-invariant direct methods
Least-squares matrix: full	Secondary atom site location: difference Fourier map
*R*[*F*^2^ > 2σ(*F*^2^)] = 0.096	Hydrogen site location: inferred from neighbouring sites
*wR*(*F*^2^) = 0.326	H atoms treated by a mixture of independent and constrained refinement
*S* = 1.06	*w* = 1/[σ^2^(*F*_o_^2^) + (0.1714*P*)^2^ + 1.0437*P*] where *P* = (*F*_o_^2^ + 2*F*_c_^2^)/3
5253 reflections	(Δ/σ)_max_ = 0.002
300 parameters	Δρ_max_ = 0.36 e Å^−^^3^
0 restraints	Δρ_min_ = −0.48 e Å^−^^3^

### Special details

**Table:**

Geometry. All e.s.d.'s (except the e.s.d. in the dihedral angle between two l.s. planes) are estimated using the full covariance matrix. The cell e.s.d.'s are taken into account individually in the estimation of e.s.d.'s in distances, angles and torsion angles; correlations between e.s.d.'s in cell parameters are only used when they are defined by crystal symmetry. An approximate (isotropic) treatment of cell e.s.d.'s is used for estimating e.s.d.'s involving l.s. planes.
Refinement. Refinement of *F*^2^ against ALL reflections. The weighted *R*-factor *wR* and goodness of fit *S* are based on *F*^2^, conventional *R*-factors *R* are based on *F*, with *F* set to zero for negative *F*^2^. The threshold expression of *F*^2^ > σ(*F*^2^) is used only for calculating *R*-factors(gt) *etc*. and is not relevant to the choice of reflections for refinement. *R*-factors based on *F*^2^ are statistically about twice as large as those based on *F*, and *R*- factors based on ALL data will be even larger.

### Fractional atomic coordinates and isotropic or equivalent isotropic displacement parameters (Å^2^)

**Table:**

	*x*	*y*	*z*	*U*_iso_*/*U*_eq_	
H1	−0.1225 (17)	0.939 (5)	0.538 (4)	0.076 (13)*	
H3	0.1457 (19)	0.725 (5)	0.070 (5)	0.090 (14)*	
H2	0.0217 (15)	1.030 (4)	0.199 (4)	0.065 (10)*	
H5	0.0956 (12)	0.980 (3)	0.076 (3)	0.049 (8)*	
H4	0.0636 (16)	0.839 (4)	0.028 (5)	0.080 (11)*	
O1	0.00828 (10)	0.7312 (2)	0.2548 (3)	0.0649 (7)	
O2	0.12260 (10)	0.8556 (2)	0.3578 (2)	0.0591 (6)	
N2	0.03006 (12)	0.9470 (3)	0.1942 (3)	0.0547 (7)	
O08	−0.19841 (12)	0.8357 (2)	0.6131 (3)	0.0756 (8)	
C2'	0.11788 (14)	0.8269 (3)	0.2236 (3)	0.0493 (7)	
N3	0.14819 (13)	0.7339 (3)	0.1580 (3)	0.0570 (7)	
C2A	0.07814 (14)	0.9016 (3)	0.1194 (3)	0.0513 (8)	
C1'	−0.00243 (15)	0.8557 (3)	0.2570 (3)	0.0556 (8)	
N1	−0.12109 (16)	0.8660 (3)	0.5058 (4)	0.0796 (11)	
O0	−0.15371 (13)	0.6498 (3)	0.5322 (3)	0.0879 (9)	
O4	0.27562 (12)	0.5598 (3)	0.2021 (3)	0.0794 (8)	
C3A	0.19058 (15)	0.6565 (3)	0.2351 (3)	0.0618 (9)	
H3A1	0.1777	0.5640	0.2558	0.074*	
H3A2	0.2000	0.7008	0.3298	0.074*	
C3'	0.24000 (15)	0.6480 (3)	0.1422 (3)	0.0576 (8)	
C1A	−0.05199 (16)	0.9118 (3)	0.3264 (4)	0.0690 (10)	
H1A1	−0.0438	1.0014	0.3701	0.083*	
H1A2	−0.0803	0.9244	0.2493	0.083*	
O3	0.24681 (12)	0.7125 (3)	0.0322 (3)	0.0932 (10)	
C02	−0.27765 (19)	0.9393 (4)	0.8130 (4)	0.0769 (11)	
H02	−0.2429	0.9693	0.8405	0.092*	
C41	0.37438 (18)	0.5699 (4)	0.2075 (4)	0.0718 (11)	
C01	−0.28408 (18)	0.8251 (4)	0.7229 (4)	0.0675 (10)	
C07	−0.23646 (18)	0.7421 (4)	0.6764 (5)	0.0773 (11)	
H07A	−0.2198	0.6951	0.7623	0.093*	
H07B	−0.2479	0.6733	0.6030	0.093*	
C0'	−0.15636 (17)	0.7739 (3)	0.5494 (4)	0.0674 (10)	
C05	−0.3807 (2)	0.8538 (5)	0.7305 (5)	0.0931 (13)	
H05	−0.4155	0.8253	0.7019	0.112*	
C1B	−0.0713 (2)	0.8209 (5)	0.4406 (5)	0.0958 (16)	
H1B1	−0.0435	0.8126	0.5196	0.115*	
H1B2	−0.0771	0.7298	0.3976	0.115*	
C42	0.3766 (2)	0.6760 (5)	0.3076 (5)	0.0898 (14)	
H42	0.3455	0.7262	0.3262	0.108*	
C47	0.32398 (19)	0.5314 (5)	0.1195 (5)	0.0872 (13)	
H47A	0.3223	0.5830	0.0264	0.105*	
H47B	0.3251	0.4338	0.0950	0.105*	
C04	−0.3732 (2)	0.9655 (5)	0.8217 (6)	0.0973 (15)	
H04	−0.4031	1.0124	0.8565	0.117*	
C03	−0.3215 (2)	1.0095 (5)	0.8629 (5)	0.0915 (13)	
H03	−0.3165	1.0865	0.9241	0.110*	
C46	0.4205 (2)	0.4963 (5)	0.1821 (6)	0.0921 (13)	
H46	0.4192	0.4236	0.1140	0.110*	
C06	−0.3362 (2)	0.7829 (4)	0.6806 (5)	0.0812 (12)	
H06	−0.3412	0.7067	0.6184	0.097*	
C44	0.4706 (3)	0.6345 (6)	0.3560 (6)	0.1102 (17)	
H44	0.5030	0.6563	0.4069	0.132*	
C45	0.4691 (2)	0.5289 (6)	0.2569 (7)	0.1087 (16)	
H45	0.5003	0.4787	0.2391	0.130*	
C43	0.4254 (3)	0.7092 (6)	0.3820 (6)	0.1114 (19)	
H43	0.4270	0.7824	0.4493	0.134*	

### Atomic displacement parameters (Å^2^)

**Table:**

	*U*^11^	*U*^22^	*U*^33^	*U*^12^	*U*^13^	*U*^23^
O1	0.0805 (17)	0.0314 (10)	0.0850 (16)	0.0001 (10)	0.0296 (13)	0.0009 (10)
O2	0.0866 (17)	0.0504 (11)	0.0408 (10)	0.0104 (11)	0.0110 (10)	−0.0026 (8)
N2	0.0747 (19)	0.0301 (11)	0.0610 (14)	0.0063 (12)	0.0226 (13)	0.0056 (10)
O08	0.0919 (19)	0.0478 (12)	0.0901 (17)	0.0053 (12)	0.0418 (15)	−0.0023 (11)
C2'	0.073 (2)	0.0336 (12)	0.0429 (13)	−0.0043 (13)	0.0195 (13)	0.0007 (10)
N3	0.080 (2)	0.0487 (14)	0.0428 (13)	0.0124 (13)	0.0082 (12)	−0.0041 (11)
C2A	0.069 (2)	0.0389 (13)	0.0473 (14)	0.0048 (14)	0.0177 (14)	0.0096 (12)
C1'	0.073 (2)	0.0342 (13)	0.0610 (16)	0.0038 (14)	0.0187 (15)	0.0019 (12)
N1	0.102 (3)	0.0498 (16)	0.091 (2)	0.0121 (17)	0.053 (2)	0.0010 (16)
O0	0.109 (2)	0.0499 (14)	0.107 (2)	0.0084 (14)	0.0342 (18)	−0.0139 (13)
O4	0.0870 (18)	0.0826 (17)	0.0705 (14)	0.0332 (15)	0.0259 (13)	0.0257 (13)
C3A	0.084 (2)	0.0523 (17)	0.0496 (15)	0.0149 (17)	0.0118 (15)	0.0027 (13)
C3'	0.076 (2)	0.0447 (15)	0.0525 (16)	−0.0003 (15)	0.0087 (15)	0.0006 (13)
C1A	0.074 (2)	0.0430 (16)	0.092 (2)	0.0066 (16)	0.0323 (19)	0.0056 (16)
O3	0.088 (2)	0.110 (2)	0.0832 (18)	0.0185 (17)	0.0207 (15)	0.0444 (17)
C02	0.092 (3)	0.060 (2)	0.080 (2)	0.000 (2)	0.022 (2)	−0.0021 (18)
C41	0.095 (3)	0.0567 (19)	0.0655 (19)	0.0159 (19)	0.027 (2)	0.0156 (16)
C01	0.087 (3)	0.0499 (17)	0.0670 (19)	−0.0021 (18)	0.0212 (18)	0.0065 (15)
C07	0.087 (3)	0.0515 (18)	0.095 (3)	0.0002 (19)	0.027 (2)	0.0023 (18)
C0'	0.091 (3)	0.0515 (18)	0.0609 (18)	0.0132 (18)	0.0239 (18)	−0.0045 (14)
C05	0.097 (3)	0.083 (3)	0.101 (3)	0.002 (3)	0.015 (3)	0.013 (3)
C1B	0.110 (4)	0.081 (3)	0.100 (3)	0.039 (3)	0.062 (3)	0.032 (2)
C42	0.113 (4)	0.071 (2)	0.088 (3)	0.011 (3)	0.034 (3)	0.003 (2)
C47	0.097 (3)	0.095 (3)	0.072 (2)	0.034 (3)	0.026 (2)	0.008 (2)
C04	0.108 (4)	0.073 (3)	0.114 (3)	0.013 (3)	0.040 (3)	0.011 (3)
C03	0.115 (4)	0.065 (2)	0.097 (3)	0.000 (3)	0.038 (3)	−0.009 (2)
C46	0.102 (4)	0.069 (2)	0.107 (3)	0.015 (3)	0.017 (3)	−0.008 (2)
C06	0.099 (3)	0.064 (2)	0.082 (2)	−0.009 (2)	0.016 (2)	−0.0005 (19)
C44	0.111 (4)	0.105 (4)	0.116 (4)	−0.017 (3)	0.023 (3)	−0.006 (3)
C45	0.094 (4)	0.092 (3)	0.140 (4)	0.012 (3)	0.011 (3)	−0.014 (3)
C43	0.140 (5)	0.090 (3)	0.108 (4)	−0.016 (3)	0.043 (4)	−0.023 (3)

### Geometric parameters (Å, º)

**Table:**

O1—C1'	1.234 (3)	C02—H02	0.9300
O2—C2'	1.234 (3)	C41—C42	1.362 (6)
N2—C1'	1.333 (4)	C41—C46	1.371 (6)
N2—C2A	1.454 (4)	C41—C47	1.495 (6)
N2—H2	0.83 (4)	C01—C06	1.389 (6)
O08—C0'	1.345 (4)	C01—C07	1.497 (5)
O08—C07	1.438 (4)	C07—H07A	0.9700
C2'—N3	1.323 (4)	C07—H07B	0.9700
C2'—C2A	1.511 (5)	C05—C04	1.362 (7)
N3—C3A	1.441 (4)	C05—C06	1.386 (6)
N3—H3	0.79 (5)	C05—H05	0.9300
C2A—H5	0.96 (3)	C1B—H1B1	0.9700
C2A—H4	1.07 (4)	C1B—H1B2	0.9700
C1'—C1A	1.496 (5)	C42—C43	1.391 (8)
N1—C0'	1.317 (5)	C42—H42	0.9300
N1—C1B	1.449 (5)	C47—H47A	0.9700
N1—H1	0.76 (4)	C47—H47B	0.9700
O0—C0'	1.214 (4)	C04—C03	1.384 (8)
O4—C3'	1.324 (4)	C04—H04	0.9300
O4—C47	1.456 (5)	C03—H03	0.9300
C3A—C3'	1.506 (5)	C46—C45	1.388 (8)
C3A—H3A1	0.9700	C46—H46	0.9300
C3A—H3A2	0.9700	C06—H06	0.9300
C3'—O3	1.183 (4)	C44—C45	1.352 (7)
C1A—C1B	1.444 (5)	C44—C43	1.359 (8)
C1A—H1A1	0.9700	C44—H44	0.9300
C1A—H1A2	0.9700	C45—H45	0.9300
C02—C03	1.369 (6)	C43—H43	0.9300
C02—C01	1.373 (5)		
			
C1'—N2—C2A	120.7 (2)	O08—C07—H07A	110.2
C1'—N2—H2	118 (3)	C01—C07—H07A	110.2
C2A—N2—H2	121 (3)	O08—C07—H07B	110.2
C0'—O08—C07	114.5 (3)	C01—C07—H07B	110.2
O2—C2'—N3	123.5 (3)	H07A—C07—H07B	108.5
O2—C2'—C2A	121.8 (3)	O0—C0'—N1	126.4 (3)
N3—C2'—C2A	114.7 (2)	O0—C0'—O08	122.7 (4)
C2'—N3—C3A	123.7 (3)	N1—C0'—O08	110.9 (3)
C2'—N3—H3	120 (3)	C04—C05—C06	119.7 (5)
C3A—N3—H3	116 (3)	C04—C05—H05	120.1
N2—C2A—C2'	112.6 (2)	C06—C05—H05	120.1
N2—C2A—H5	109.7 (18)	C1A—C1B—N1	114.3 (3)
C2'—C2A—H5	109.6 (18)	C1A—C1B—H1B1	108.7
N2—C2A—H4	105 (2)	N1—C1B—H1B1	108.7
C2'—C2A—H4	113 (2)	C1A—C1B—H1B2	108.7
H5—C2A—H4	106 (3)	N1—C1B—H1B2	108.7
O1—C1'—N2	120.5 (3)	H1B1—C1B—H1B2	107.6
O1—C1'—C1A	122.7 (3)	C41—C42—C43	120.1 (5)
N2—C1'—C1A	116.8 (3)	C41—C42—H42	120.0
C0'—N1—C1B	119.8 (3)	C43—C42—H42	120.0
C0'—N1—H1	118 (3)	O4—C47—C41	111.7 (3)
C1B—N1—H1	119 (3)	O4—C47—H47A	109.3
C3'—O4—C47	117.6 (3)	C41—C47—H47A	109.3
N3—C3A—C3'	110.8 (3)	O4—C47—H47B	109.3
N3—C3A—H3A1	109.5	C41—C47—H47B	109.3
C3'—C3A—H3A1	109.5	H47A—C47—H47B	107.9
N3—C3A—H3A2	109.5	C03—C04—C05	120.4 (5)
C3'—C3A—H3A2	109.5	C03—C04—H04	119.8
H3A1—C3A—H3A2	108.1	C05—C04—H04	119.8
O3—C3'—O4	124.2 (3)	C04—C03—C02	119.7 (4)
O3—C3'—C3A	125.1 (3)	C04—C03—H03	120.2
O4—C3'—C3A	110.7 (3)	C02—C03—H03	120.2
C1B—C1A—C1'	111.8 (3)	C41—C46—C45	120.7 (5)
C1B—C1A—H1A1	109.3	C41—C46—H46	119.7
C1'—C1A—H1A1	109.3	C45—C46—H46	119.7
C1B—C1A—H1A2	109.3	C01—C06—C05	120.4 (4)
C1'—C1A—H1A2	109.3	C01—C06—H06	119.8
H1A1—C1A—H1A2	107.9	C05—C06—H06	119.8
C03—C02—C01	121.1 (5)	C45—C44—C43	120.8 (6)
C03—C02—H02	119.5	C45—C44—H44	119.6
C01—C02—H02	119.5	C43—C44—H44	119.6
C42—C41—C46	119.2 (5)	C44—C45—C46	119.3 (6)
C42—C41—C47	123.1 (4)	C44—C45—H45	120.3
C46—C41—C47	117.6 (4)	C46—C45—H45	120.3
C02—C01—C06	118.7 (4)	C44—C43—C42	119.9 (5)
C02—C01—C07	121.4 (4)	C44—C43—H43	120.1
C06—C01—C07	119.8 (3)	C42—C43—H43	120.1
O08—C07—C01	107.7 (3)		
			
O2—C2'—N3—C3A	−0.8 (5)	C07—O08—C0'—O0	7.7 (6)
C2A—C2'—N3—C3A	176.5 (3)	C07—O08—C0'—N1	−174.6 (4)
C1'—N2—C2A—C2'	−61.0 (4)	C1'—C1A—C1B—N1	−176.6 (4)
O2—C2'—C2A—N2	−31.3 (4)	C0'—N1—C1B—C1A	146.5 (5)
N3—C2'—C2A—N2	151.4 (3)	C46—C41—C42—C43	0.4 (6)
C2A—N2—C1'—O1	1.2 (5)	C47—C41—C42—C43	−177.8 (4)
C2A—N2—C1'—C1A	−177.8 (3)	C3'—O4—C47—C41	119.7 (4)
C2'—N3—C3A—C3'	−137.2 (3)	C42—C41—C47—O4	−31.4 (5)
C47—O4—C3'—O3	−5.8 (6)	C46—C41—C47—O4	150.4 (4)
C47—O4—C3'—C3A	175.3 (3)	C06—C05—C04—C03	0.9 (7)
N3—C3A—C3'—O3	10.8 (5)	C05—C04—C03—C02	−0.8 (7)
N3—C3A—C3'—O4	−170.4 (3)	C01—C02—C03—C04	−0.2 (7)
O1—C1'—C1A—C1B	25.1 (6)	C42—C41—C46—C45	−0.1 (7)
N2—C1'—C1A—C1B	−155.9 (4)	C47—C41—C46—C45	178.2 (4)
C03—C02—C01—C06	1.1 (6)	C02—C01—C06—C05	−1.0 (6)
C03—C02—C01—C07	−175.8 (4)	C07—C01—C06—C05	176.0 (4)
C0'—O08—C07—C01	−172.3 (3)	C04—C05—C06—C01	0.0 (6)
C02—C01—C07—O08	−52.3 (5)	C43—C44—C45—C46	−0.5 (9)
C06—C01—C07—O08	130.8 (4)	C41—C46—C45—C44	0.1 (8)
C1B—N1—C0'—O0	−5.3 (7)	C45—C44—C43—C42	0.9 (9)
C1B—N1—C0'—O08	177.1 (4)	C41—C42—C43—C44	−0.8 (7)

### Hydrogen-bond geometry (Å, º)

**Table:**

*D*—H···*A*	*D*—H	H···*A*	*D*···*A*	*D*—H···*A*
N2—H2···O1^i^	0.83 (4)	2.13 (4)	2.951 (3)	169 (4)
N3—H3···O2^ii^	0.79 (5)	2.11 (5)	2.868 (3)	161 (5)
N1—H1···O2^iii^	0.76 (5)	2.20 (5)	2.959 (4)	176 (2)

Symmetry codes: (i) −*x*, *y*+1/2, −*z*+1/2; (ii) *x*, −*y*+3/2, *z*−1/2; (iii) −*x*, −*y*+2, −*z*+1.
