# Supplementary material for: The tripeptide N-Cbz-βGly-Gly-Gly-Obz
Source: Acta Crystallogr E Crystallogr Commun. 2015 Mar 14;71(Pt 4):o240–1. doi: 10.1107/S2056989015004272 (PMC4438843; doi:10.1107/S2056989015004272)
Supplement: Supplementary file 3 [file e-71-0o240-Isup3.docx]

**Comment**

Insertion of methylene units to the backbone of α-amino acids generates a family of amino acids referred to as ω-amino acids (Cheng *et al*., 2001; Seebach *et al.*, 2004). β-amino acids are obtained when a single methylene unit is added to the backbone of an α-amino acid. Compounds containing β-amino acids are ubiquitously found in biological systems (Seebach *et al.*, 2004). The simplest β-amino acid, β-glycine, is a component of co-enzyme A, pantothenic acid and carnosine. β-amino acids have an additional degree of torsional freedom about the C^β^ - C^α^ bond (θ) and this increases the conformational possibilities of peptides formed of β-amino acids.

Interest in β-amino acids has resulted in a considerable body of work on the conformation of polypeptides formed of β-amino acids (Seebach and Matthews, 1997; Gellman, 1998; Hill *et al.*, 2001; Cheng *et al.*, 2001; Seebach *et al.*, 2004, 2005, 2006). Hybrid sequences containing α-, β- and higher ω-amino acids have also been investigated (Banerjee and Balaram, 1997; Karle *et al.*, 1997; Gopi *et al.*, 2002; Roy and Balaram, 2004; Ananda *et al.*, 2005; Roy *et al.*, 2005; Schmitt *et al.*, 2005, 2006; Sharma *et al.*, 2009; Schramm *et al.*, 2010). Helical structures formed by β-peptides have been observed by several research groups (Seebach *et al.*, 1996, 2005; Appella *et al.*, 1996, 1997).

In case of β-amino acids, information regarding the conformational preferences can only be obtained by crystallographic characterization of synthetic peptides unlike in case of α-amino acids where such information can be gathered from the crystal structures of proteins. This paper presents the crystallographic characterization of a synthetic peptide containing a β-glycine residue.


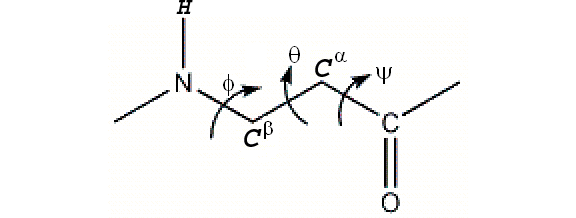


**Figure 3**: Atomic labeling and definition of backbone torsion angles in case of β-residues.

**Experimental**

The title compound was purchased commercially. Plate-like crystals of the title compound were obtained by slow evaporation from methanol/water solution.

**Refinement**

The N-bound H atoms and H-atoms bound to C2A could be located from difference Fourier maps. The remaining C-bound H atoms were fixed geometrically in calculated positions and refined as riding atoms. During refinement, H-atoms attached to aromatic rings were positioned with C-H = 0.93Å and U_iso_(H) = 1.2 U_eq_ while methylene H-atoms were positioned with C-H = 0.97Å and U_iso_(H) = 1.2 U_eq_.

**Results and Discussion**

**Molecular Conformation**

The first two glycine residues of the peptide molecule adopt extended conformations while the third glycine residue adopts a helical conformation. βGly(1) adopts torsion angle values *φ*_1_ = 146.5° and *ψ*_1_ = -155.9°. *Trans* conformation is observed about the C^β^ - C^α^ bond of the βGly(1) residue. Gly(2) adopts torsion angles *φ*_2_ = -61.0° and *ψ*_2_ = 151.4° while Gly(3) adopts torsion angle values *φ*_3_ = -137.2° and *ψ*_3_ = -170.4°. Since the crystal structure is that of an achiral peptide crystallized in a centrosymmetric space group, the choice of sign for torsion angles is arbitrary. There are no intramolecular hydrogen bonds in the crystal structure.

**Supramolecular features**

In the crystal, three N—H···O intermolecular hydrogen bonds, one of them bifurcated involving a carbonyl oxygen and two donor NH , results in an infinite two-dimensional network parallel to the *bc* plane.


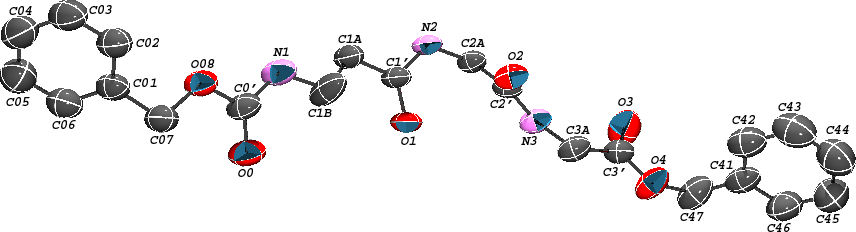


**Figure 1**: Thermal Ellipsoid plot of **NCbz-βGly-Gly-Gly-Obz** drawn at 50% probability level. Hydrogen atoms have been omitted for clarity.


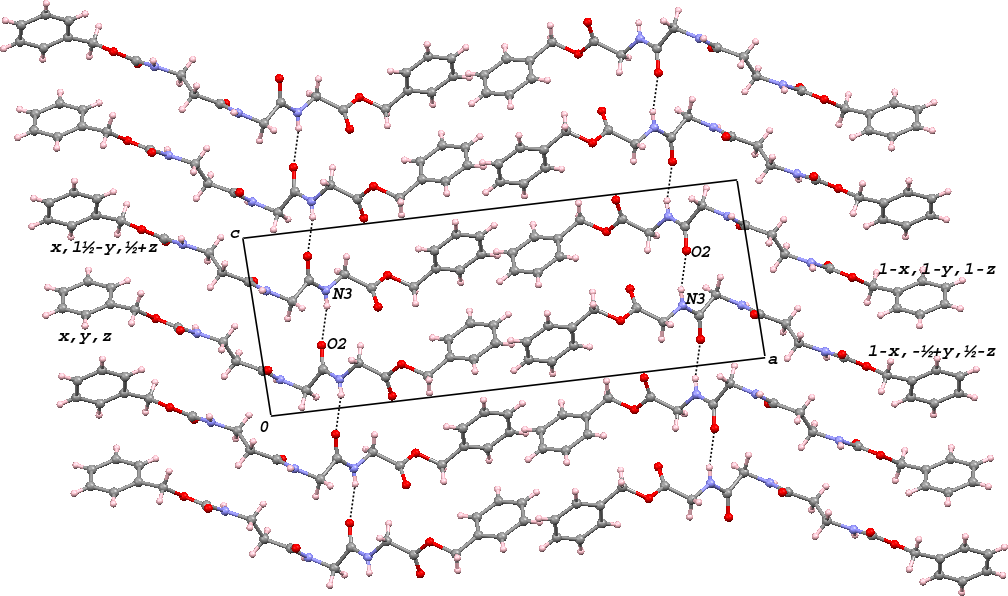


**Figure 2**: A view of the packing of **NCbz-βGly-Gly-Gly-Obz** as viewed down the b-axis. Intermolecular hydrogen bonds are represented as dotted lines.
