# Supplementary figures and images for: The tripeptide N-Cbz-βGly-Gly-Gly-Obz
Source: Acta Crystallogr E Crystallogr Commun. 2015 Mar 14;71(Pt 4):o240–1. doi: 10.1107/S2056989015004272 (PMC4438843; doi:10.1107/S2056989015004272)

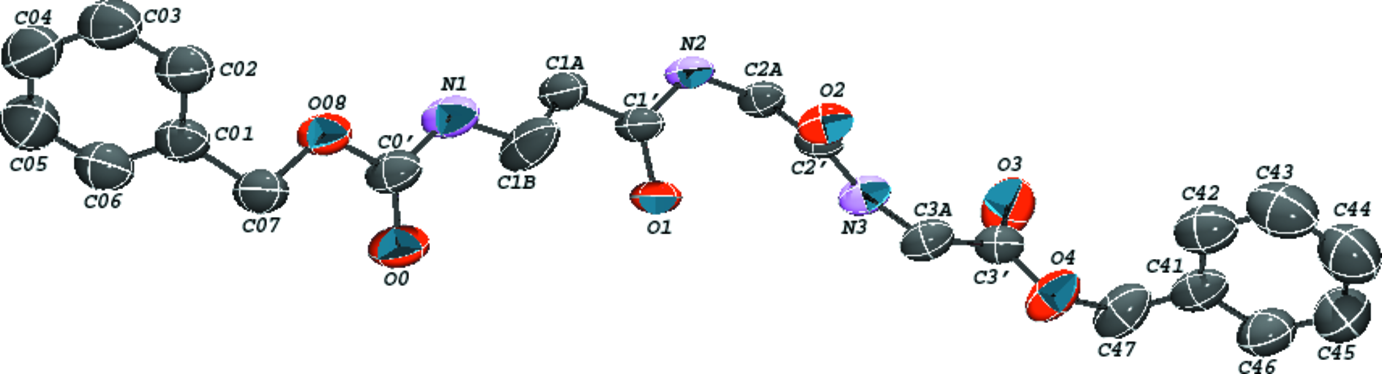

Supplement: Supplementary file 5 [file e-71-0o240-fig1.tif]

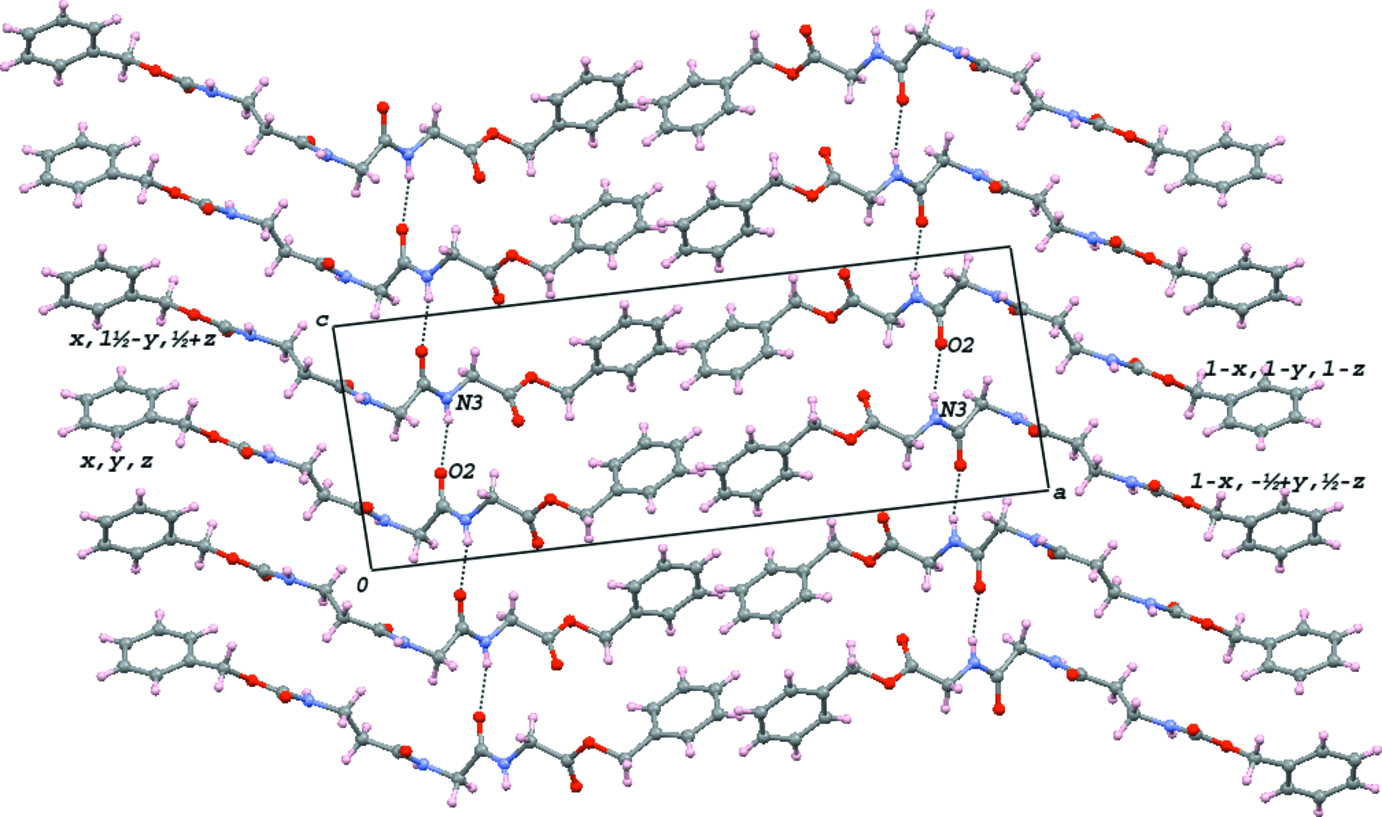

Supplement: Supplementary file 6 [file e-71-0o240-fig2.tif]

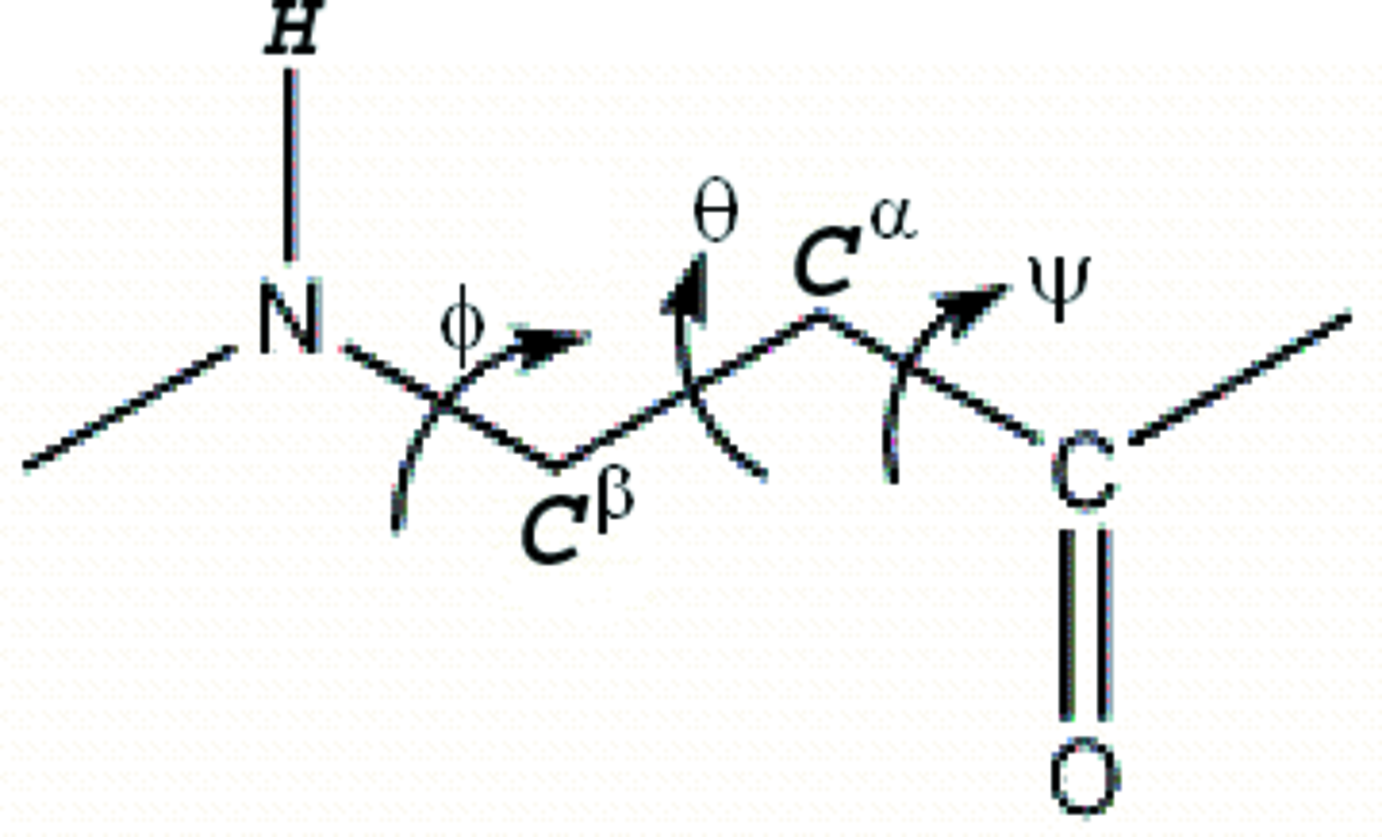

Supplement: Supplementary file 7 [file e-71-0o240-fig3.tif]
